# Occurrence and Fate of Substituted *p*-Phenylenediamine-Derived Quinones in Hong Kong Wastewater Treatment Plants

**DOI:** 10.1021/acs.est.3c03758

**Published:** 2023-10-05

**Authors:** Guodong Cao, Wei Wang, Jing Zhang, Pengfei Wu, Han Qiao, Huankai Li, Gefei Huang, Zhu Yang, Zongwei Cai

**Affiliations:** State Key Laboratory of Environmental and Biological Analysis, Department of Chemistry, Hong Kong Baptist University, Hong Kong SAR999077, China

**Keywords:** *para*-phenylenediamines, rubber-derived quinones, wastewater treatment plants, removal efficiency, mass balance

## Abstract

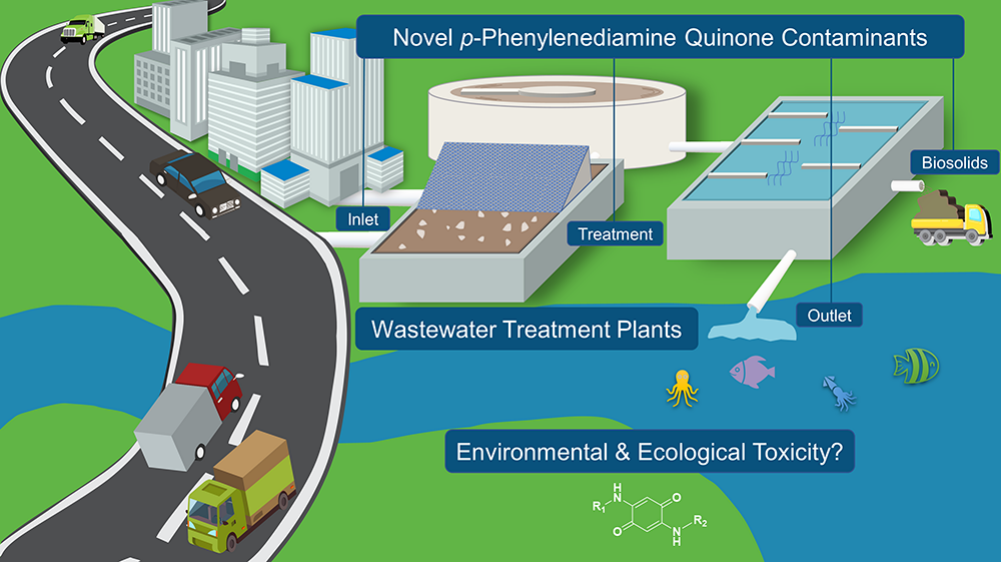

*para*-Phenylenediamine quinones (PPD-Qs) are a newly discovered class of transformation products derived from *para*-phenylenediamine (PPD) antioxidants. These compounds are prevalent in runoff, roadside soil, and particulate matter. One compound among these, *N*-1,3-dimethylbutyl-*n*′-phenyl-*p*-phenylenediamine quinone (6PPD-Q), was found to induce acute mortality of coho salmon, rainbow trout, and brook trout, with the median lethal concentrations even lower than its appearance in the surface and receiving water system. However, there was limited knowledge about the occurrence and fate of these emerging environmental contaminants in wastewater treatment plants (WWTPs), which is crucial for effective pollutant removal via municipal wastewater networks. In the current study, we performed a comprehensive investigation of a suite of PPD-Qs along with their parent compounds across the influent, effluent, and biosolids during each processing unit in four typical WWTPs in Hong Kong. The total concentrations of PPDs and PPD-Qs in the influent were determined to be 2.7–90 and 14–830 ng/L. In the effluent, their concentrations decreased to 0.59–40 and 2.8–140 ng/L, respectively. The median removal efficiency for PPD-Qs varied between 53.0 and 91.0% across the WWTPs, indicating that a considerable proportion of these contaminants may not be fully eliminated through the current processing technology. Mass flow analyses revealed that relatively higher levels of PPD-Qs were retained in the sewage sludge (20.0%) rather than in the wastewater (16.9%). In comparison to PPDs, PPD-Qs with higher half-lives exhibited higher release levels via effluent wastewater, which raises particular concerns about their environmental consequences to aquatic ecosystems.

## Abstract 2

PPDs-derived quinones are a new and unexpected class of organic contaminant found in Hong Kong four WWTPs; these compounds cannot be fully eliminated through current processing technologies with a subsequent discharge into natural water bodies via treated municipal wastewater at the ng/L level.

## 1 Introduction

Substituted *para*-phenylenediamines (PPDs) are synthetic antioxidants that are widely used in the rubber industry for the production of tires, hoses, belts, and shoe soles.^1−3^ The widespread use of these chemicals has led to their massive production and consumption worldwide, and some of them have been listed as high production volume chemicals by the U.S. EPA in 2015,^4^ including *N*-1,3-dimethylbutyl-*n*′-phenyl-*p*-phenylenediamine (6PPD, 50–100 million lbs/year), *N*,*N*′-diphenyl-*p*-phenylenediamine (DPPD, <1 million lbs/year), *N*-isopropyl-*m*′-phenyl-*p*-phenylenediamine (IPPD, 115 thousand lbs/year), and *N*,*N*′-bis(methylphenyl)-1,4-benzenediamine (DTPD, 61 thousand lbs/year). In China, the production of PPDs was approximately 0.1 million tons in 2009, while the volume increased gradually to more than 0.2 million tons in 2020 owing to the high and global rubber consumption.^5,6^ As a consequence, PPD-associated contaminants have become pervasive in our environment,^7,8^ potentially causing adverse effects on living organisms, including humans, rats, herbivores, and aquatic species.^9−11^

As published in Science in 2020, Tian and colleagues identified a new environmental transformation product of 6PPD, named 6PPD-Q, present in tire rubber leachate and urban watersheds.^12^ The toxicant was found to induce acute mortality of Pacific Northwest coho salmon (*Oncorhynchus kisutch*) with a relatively low median lethal concentration (LC_50_, 95 ng/L).^13^ This value was substantially lower than the lethality threshold measured for its parent compound 6PPD (LC_50_, 250 μg/L).^12,13^ Subsequent studies have demonstrated that other aquatic species, such as rainbow trout and brook trout are exceptionally susceptible to this contaminant, which aroused particular concerns for interrogating its occurrence in the aquatic environment worldwide. Available data suggested that concentrations of 6PPD-Q in urban surface water of the U.S. varied within the range of <300–3500 ng/L,^12^ while its concentrations in the creek of Canada were determined to be 210–760 ng/L.^14^ Both exceeded the LC_50_ values of 6PPD-Q for coho salmon. The contaminant was also detected in snowmelt samples in a cold-climate city of Saskatoon in Canada with mean concentrations of 80–370 ng/L.^15^ Another study conducted by Seiwert et al. indicated that 6PPD-Q was detectable in municipal wastewater of Leipzig, Germany with measured levels varying from <25 to 105 ng/L.^16^ New evidence suggests that not only 6PPD-Q, but also other PPD-derived quinones (PPD-Qs) are ubiquitously present in our environment. Considerable levels of these contaminants have been detected in the urban runoff, roadside soil, particulate matter, e-waste dust, and sediments across rivers, estuaries, and deep-sea regions.^17−24^ In a recent study, Wang et al. demonstrated that these newly discovered contaminants are redox-active species and greatly contribute to the oxidative potential of particulate matter in different megacities in China.^25^ Current findings collectively indicated that the widespread occurrence of these emerging contaminants in terrestrial and aquatic environments is closely related to anthropogenic activities.

Wastewater treatment plants (WWTPs) represent a source of anthropogenic chemical release into the aquatic environment. This is due in large part to the diverse origins of the sewage influent, which can include municipal and industrial discharges, urban runoff, and the residues of consumer products.^26,27^ As PPD-Qs and their parent compounds PPDs were found to be prevalent in the environment, particularly in urban runoff and stormwater,^28,29^ such phenomena lead us to postulate that quite an amount of these chemicals may end up in WWTPs through the municipal sewage network. A recent study has indicated that 6PPD-Q was detectable in urban streams and WWTP discharge points in the Greater Toronto Area during a period of dry weather.^14^ However, little information is currently available about the occurrence and composition profiles of these emerging contaminants in urban WWTPs with different treatment techniques. The removal behaviors of PPD-Qs and their parent PPDs during each processing unit in WWTPs remain elusive.

Our current research focuses on interrogating the prevalence and fate of PPD-Qs in four typical WWTPs in Hong Kong with different treatment systems. Using self-synthesized standards, the concomitance of PPD-Qs and their parent PPDs in the influent, effluent, and biosolids during each processing unit was determined, and the removal efficiency of these contaminants was assessed. In particular, mass flow and mass balance analyses were conducted to facilitate a more thorough understanding of the transport, removal mechanisms, and environmental releases of these compounds.

## 2 Materials and Methods

### 2.1 Chemicals and Reagents

PPDs standards (Table S1) with purities >98% were purchased from J&K Chemical Ltd. (Hong Kong, China) and TCI Chemicals (Tokyo, Japan). 6PPD-Q (purity, 95%) was obtained from Cambridge Isotope Laboratories. The four other PPD-Qs as listed in Table S1 and the deuterated 6PPD-Q-*d*_5_ were synthesized according to our previously published protocols.^21^ The surrogate standard diphenylamine-d_10_ was purchased from TRC (Burlington, Canada). The purities of PPD-quinone standards were estimated to be higher than 95% based on their integrated ^1^H nuclear magnetic resonance (NMR) spectra.

### 2.2 Sample Collection

Wastewater and biosolid samples were collected from four WWTPs located in Hong Kong, which collectively serve a population of 4.4 million (approximately 60%) of the city’s residents in 2021. Their operational characteristics, including the daily flows and catchment populations, are summarized in Table S2. Plant Stonecutters Island (SI) is the largest WWTP in Hong Kong where its raw wastewater is collected from seven preliminary treatment works from Kowloon and Hong Kong Island. Plant SI and Plant Siu Ho Wan (SHW) both utilize chemically enhanced primary treatment (CEPT) for rapid sedimentation, with ferric chloride and polymers being added. Plants Sha Tin (ST) and Stanley (SL) are secondary WWTPs using activated sludge processes and either an anaerobic/oxic (A/O) or moving-bed biofilm reactor (MBBR) for treatment of wastewater. Sampling campaigns were conducted during weekdays from October 11 to November 18, 2021. Using a poly(methyl methacrylate) (PMMA) water collector, an automated sampling device was used to collect the successive 24 h composite samples with a sampling interval of 1 h. The composite samples are time-weighted, and the hydraulic retention time on water phases is less than 24 h. The sampling campaigns were conducted twice for each processing unit. Each batch of wastewater was collected for 2 L, while 500 g of biosolid samples was obtained at each WWTP for analysis. The flows were measured by the investigated WWTPs during the sampling period, and the result was comparable to their annual average flows. Biosolids were collected after the dewatering process using a stainless-steel shovel. Both the wastewater and biosolids were collected in glass bottles that had been washed with deionized water (Milli-Q) and methanol before being used. The collected wastewater (*n* = 40) and biosolid (*n* = 8) samples were immediately transferred into ice coolers and transported to the laboratory within 2 h. Once in the laboratory, the wastewater was filtered through a glass microfiber filter (1.2 μm, Whatman, Hillsboro, USA) for removal of suspended particulate matter. The filtrate was added with 5% (v/v) methanol for inhibiting microbial growth and then stored in the dark at 4 °C for sample extraction.^30^ The biosolids and filtered suspended particulate matter samples were freeze-dried, homogenized, passed through a 60-mesh sieve, and stored at −20 °C before use.

### 2.3 Sample Extraction

Processing and treatment of wastewater and biosolids followed published approaches with modifications.^21,31^ Generally, appropriate volumes (250 mL for influent wastewater, diluted with 250 mL of deionized water to reduce matrix effects; 500 mL for other wastewater samples) of water samples spiked with 50 ng of surrogate standard were consecutively extracted three times using 50, 25, and 25 mL of dichloromethane, respectively. The combined organic extracts were concentrated to 1 mL of the mixture under nitrogen. Purification was performed on an Envi-carb SPE cartridge, which was eluted with 3 mL of methanol/dichloromethane (2:8, v/v) at a flow rate of 0.8 mL/min. After that, the elutes were nitrogen purged to near dryness and reconstituted in 500 μL acetonitrile containing 20 ng of 6PPD-Q-*d*_5_ (internal standard). The samples were filtered through a nylon filter membrane (Navigator, 0.45 μm) prior to instrument analysis. For biosolids, 100 mg of samples was spiked with 20 ng of surrogate standard and ultrasonically extracted two times with 3 mL of dichloromethane (15 min each). The residues were extracted for another 15 min with 3 mL of acetonitrile. The extracts were then combined and concentrated to 1 mL, followed by the SPE cartridge cleanup procedure as described above. The eluates were dried to dryness, redissolved in 500 μL of acetonitrile containing 20 ng of 6PPD-Q-*d*_5_, and filtered for instrument analysis.

### 2.4 Instrumental Analysis

Sample extracts were analyzed using a Thermo Vanquish MD HPLC coupled to a triple quadrupole mass spectrometer (Altis, ThermoFisher, US). Chromatographic separation of PPD-Qs and PPDs was performed using a Waters Acquity HSS T3 column (1.8 μm, 2.1 × 100 mm), where the mobile phase consisted of 0.1% formic acid in deionized water (A) and 0.1% formic acid in acetonitrile (B). Gradient elution (300 μL/min) was as follows: initial with 2% phase B for 1 min, then gradually increased to 100% phase B in 19 min, held for 3 min, finally decreased to 2% phase B in 0.1 min, and held for another 4.9 min. Analytes were monitored by using the multiple reaction monitoring (MRM) mode. Collision energies for each precursor/product ion pair were optimized and are listed in Table S3.

### 2.5 Quality Control and Assurance

Serially diluted standard solutions with an internal standard were prepared for constructing calibration curves. To monitor the possible influence from carryover, background contamination, and system performance, a field blank, procedure blank, and an independent check standard (20 μg/L) were processed sequentially for each batch of samples. Field blank samples with water and biosolids free of analytes were parallelly carried with the collected samples to assess whether contamination may have occurred during sampling. The stability and half-lives of PPD-Qs and PPDs in dechlorinated tap water were determined, and the details are described in Text S1 of Supporting Information. In the field blanks, no targeted analytes were found, and the abundance variation of the check standards fell within acceptable ranges (<20%). To examine the recovery of the method, 50 ng of PPDs and PPD-Qs were spiked into 100 mg of biosolids and 500 mL wastewater samples, respectively, which were processed through the entire extraction procedure in triplicate. The recoveries of analytes were defined as the ratio of measured (subtracted to the background value) and spiked concentrations, which were determined to be 71 ± 6 to 111 ± 11% in biosolids and 75 ± 16 to 113 ± 22% in wastewater (Table S3). Method reproducibility was evaluated using a triplicate analysis of a blank sample spiked with mixed 10 standards with a concentration of 50 μg/L, where the relative standard deviation (RSD) was found to be satisfactory with all values falling below 15%. The determination of LOD and LOQ is based on the S/N method by spiking mixed standards in the blank environmental sample (rainwater was collected from a rainwater tank in Plant SI, which was exposed to the blazing sun with no targeted analytes being detected) and diluting it until the S/N of the chromatography peak is 3 and 10 times higher than the baseline.

### 2.6 Data Analysis

The MS data were extracted and processed using Xcalibur software (Thermo Fisher, USA). Estimation Programs Interface (EPI) Suite (US EPA, Version 4.1) was used for estimation of the physicochemical properties of PPD-Qs and PPDs (Table S1). Nonparametric methods were adopted using the Statistical Program for Social Sciences (SPSS, Version 24.0, IBM, SPSS Inc.) software. Statistical significance was assumed if the *p* value was less than 0.05. Calculations of the parameters, including removal efficiencies, mass fluxes, mass balance, and emission mass loads, are described in Text S2 of the Supporting Information.

## 3 Results and Discussion

### 3.1 Occurrence and Composition Profiles of PPD-Qs and PPDs in WWTPs

Table 1 illustrates the concentrations and detection frequencies of five PPD-Qs, along with their parent PPDs in the influent, effluent, and biosolids, among the four investigated Hong Kong WWTPs. All of the target compounds, except DTPD-Q and DTPD, exhibited detection frequencies greater than 75% in all measured samples. The occurrence of CPPD-Q, DPPD-Q, and IPPD-Q was reported for the first time in both wastewater and biosolids. These findings support the presence of a suite of prevalent but previously overlooked PPD-Q contaminants in Hong Kong’s municipal wastewater systems. In parallel, we have measured the half-lives of PPD-Qs and PPDs in dechlorinated tap water (Table S1). The results suggest that PPD-Qs exhibit longer half-lives than the corresponding PPDs, indicating the relative stability of these contaminants in aqueous systems (Figure S1). The median concentrations of PPD-Qs in the influent ranged from 0.20 to 110 ng/L, while their parent PPDs varied from 0.35 to 12 ng/L. Notably, DPPD-Q (median of 110 ng/L) and 6PPD-Q (median of 53 ng/L) were the dominant species among the PPD-Qs in the influent, followed by IPPD-Q and CPPD-Q. This is in line with the production volume of PPDs, accompanying their high-frequency usage in rubber-related products.^4,6^ As compared, the median concentrations of DPPD (0.56 ng/L) and DTPD (0.35 ng/L) in the Hong Kong WWTPs were slightly lower than their reported concentrations in Canadian WWTPs, which were 0.83 and 0.79 ng/L, respectively.^31^ In addition, considerable levels of PPD-Qs and PPDs were found to be retained in the effluent. A similar congener profile was observed for PPD-Qs in both the influent and effluent, where DPPD-Q was identified as the dominant compound, followed by 6PPD-Q, IPPD-Q, and CPPD-Q. In contrast, IPPD exhibited the highest concentrations in the effluent, followed by 6PPD, DPPD, CPPD, and DTPD. The median concentrations of PPD-Qs in the wastewater effluent were determined in the range of 0.04 to 4.3 ng/L, which were higher than their parent PPDs (<LOQ to 0.71 ng/L). These findings suggest that current processing technologies in the investigated Hong Kong WWTPs cannot completely eliminate these contaminants, especially PPD-Qs. As a consequence, these contaminants are being discharged into the receiving water bodies via treated municipal wastewater at the ng/L level.

**Table 1:** Detection Frequencies and Concentrations of PPDs and PPD-Qs in Wastewater (ng/L) and Biosolids (ng/g) in Hong Kong WWTPs

compounds	influent (ng/L)			effluent (ng/L)			biosolids (ng/g)		
	median	range	DF^a^	median	range	DF	median	range	DF
IPPD	5.5	0.63–33	100	0.71	0.13–28	100	0.47	0.25–1.9	100
CPPD	0.40	<LOQ^b^-1.2	100	0.13	0.05–0.2	100	0.65	0.48–0.83	100
6PPD	12	1.1–59	100	0.30	<LOQ-15	100	5.5	2.1–71	100
DPPD	0.56	0.39–1.2	100	0.20	<LOQ-0.28	88	0.64	0.49–2.0	100
DTPD	0.35	<LOQ-1.3	50	<LOQ	<LOQ-0.3	38	0.54	0.53–0.74	100
ΣPPDs	21	2.7–90	100	1.7	0.59–40	100	7.9	3.9–80	100
IPPD-Q	0.96	0.36–3.5	100	0.41	0.06–1.7	100	0.19	<LOQ-0.39	100
CPPD-Q	0.20	<LOQ-0.36	75	0.04	<LOQ-0.16	75	1.2	0.35–2.5	100
6PPD-Q	53	1.9–470	100	3.4	1.1–37	100	6.4	2.6–7.3	100
DTPD-Q	NC^c^	ND^d^	0	NC^c^	ND^d^	0	NC^c^	ND^d^	0
DPPD-Q	110	11–360	100	4.3	1.1–100	100	45	19–240	100
ΣPPD-Qs	170	14–830	100	7.8	2.8–140	100	53	22–250	100

a DF: defection frequency (%).

b LOQ: limit of quantification.

c NC, not calculated.

d ND, not detected.

Our survey also provides evidence for the concomitant PPD-Qs and PPDs in the biosolids. The concentrations of PPD-Qs were determined with a decreasing order of DPPD-Q (median of 45 ng/g), 6PPD-Q (median of 6.4 ng/g), CPPD-Q (median of 1.2 ng/g), and IPPD-Q (median of 0.19 ng/g). Being the most dominant compounds in biosolids, the levels of DPPD-Q and 6PPD-Q were found to be comparable to other well-known contaminants, such as antiviral drugs and brominated flame retardants^32,33^ but lower than those of benzotriazoles, polychlorinated biphenyls, and perfluoroalkyl and polyfluoroalkyl substances.^34−36^ Similar to the results observed in the influent and effluent, the median concentrations of ΣPPD-Qs (median of 53 ng/L, range of 22–250 ng/L) determined in biosolids were 6.7-fold higher than that of ΣPPDs (median of 7.9 ng/L, range of 3.9–80 ng/L). Since a certain amount of the biosolids generated from Hong Kong WWTPs was recycled as a soil conditioner,^37^ special attention should be paid to the repurposed biosolids containing these contaminants in agriculture and soil amendment.

**Figure 1:**
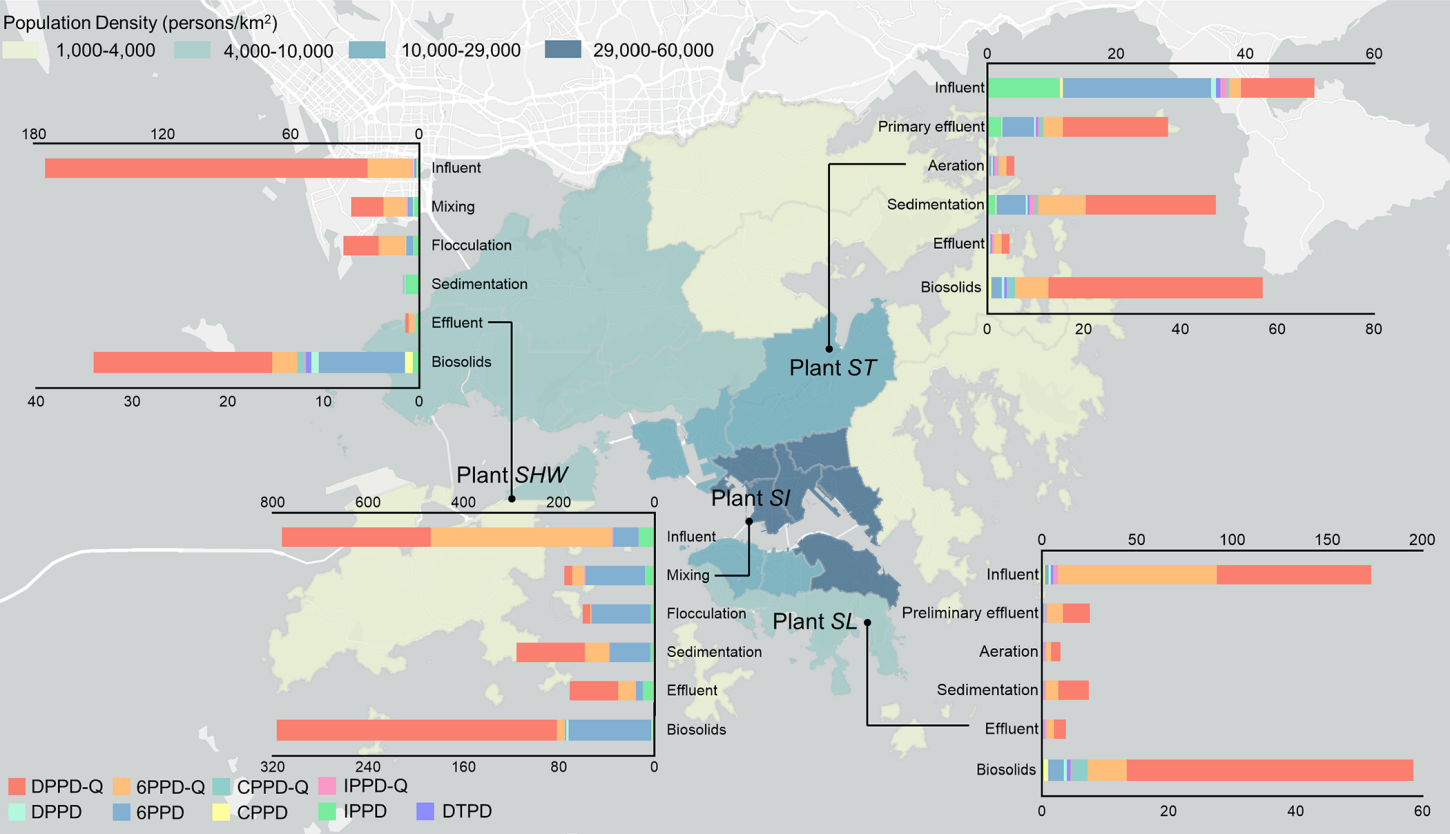
Compositions and mean concentrations (ng/L in wastewater and ng/g in biosolids) of PPD-Qs and PPDs in each processing unit of Hong Kong WWTPs.

For a better understanding of the occurrence of PPD-Qs and PPDs in each processing unit, their mean concentrations and composition profiles among the four WWTPs are depicted in Figure 1. As the largest WWTP in Hong Kong, Plant SI receives wastewater from seven preliminary treatment works at Kowloon and northeastern Hong Kong Island (Figure S2), covering approximately 47% of the total population in Hong Kong. A significant amount of PPDs and PPD-Qs (780 ± 140 ng/L) was emitted via the wastewater due to vehicle emissions, and living and commercial activities in this area.^38^ Plant SHW exhibited the second-highest concentrations of PPDs and PPD-Qs in the influent (180 ± 14 ng/L), slightly higher than those of Plant SL (170 ± 1.2 ng/L). Both WWTPs are situated on an island and are primarily surrounded by tourist areas, including amusement parks (Figure S2). In contrast, Plant ST, which is located in Ma Liu Shui, a predominantly residential area of Hong Kong, had the lowest influent concentrations of PPDs and PPD-Qs (51.0 ± 0.37 ng/L). Our results collectively indicated that among the four investigated WWTPs, most PPD-Qs exhibited a higher input amount than their parent PPDs, with DPPD-Q and 6PPD-Q being the most dominant compounds. These findings also demonstrated that the levels of PPD-Qs in the influent of each WWTP were apparently lower than their levels measured in the roadway runoff at the same city.^21^ This can be rationalized by the dilution of industrial wastewater, domestic wastewater, and rainfall convergence.

### 3.2 Removal Efficiency and Mass Balance of PPD-Qs and PPDs in WWTPs

**Figure 2:**
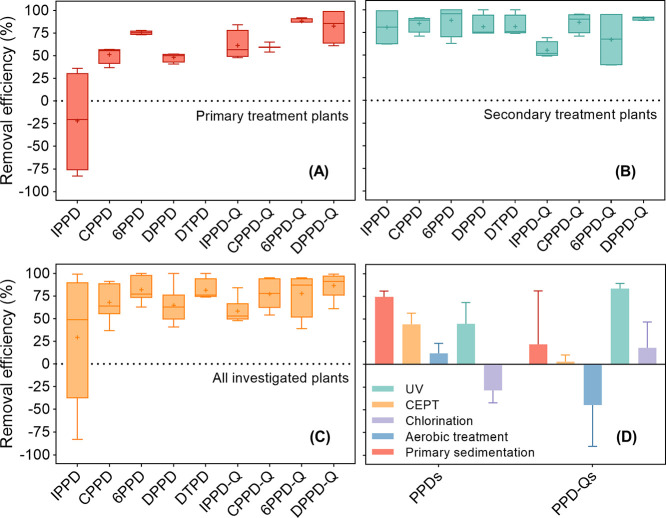
Removal efficiencies of PPD-Qs and PPDs in Plants ST and SL (A). Plants of SI and SHW (B) among all the studied WWTPs (C) with different treatment systems (D). Each water sample was collected twice, as the results in the graph are the average of the two measurements. Error bars represent the standard deviations of samples grouped by either primary treatment plants/secondary treatment plants or different processing techniques among the investigated WWTPs.

**Figure 3:**
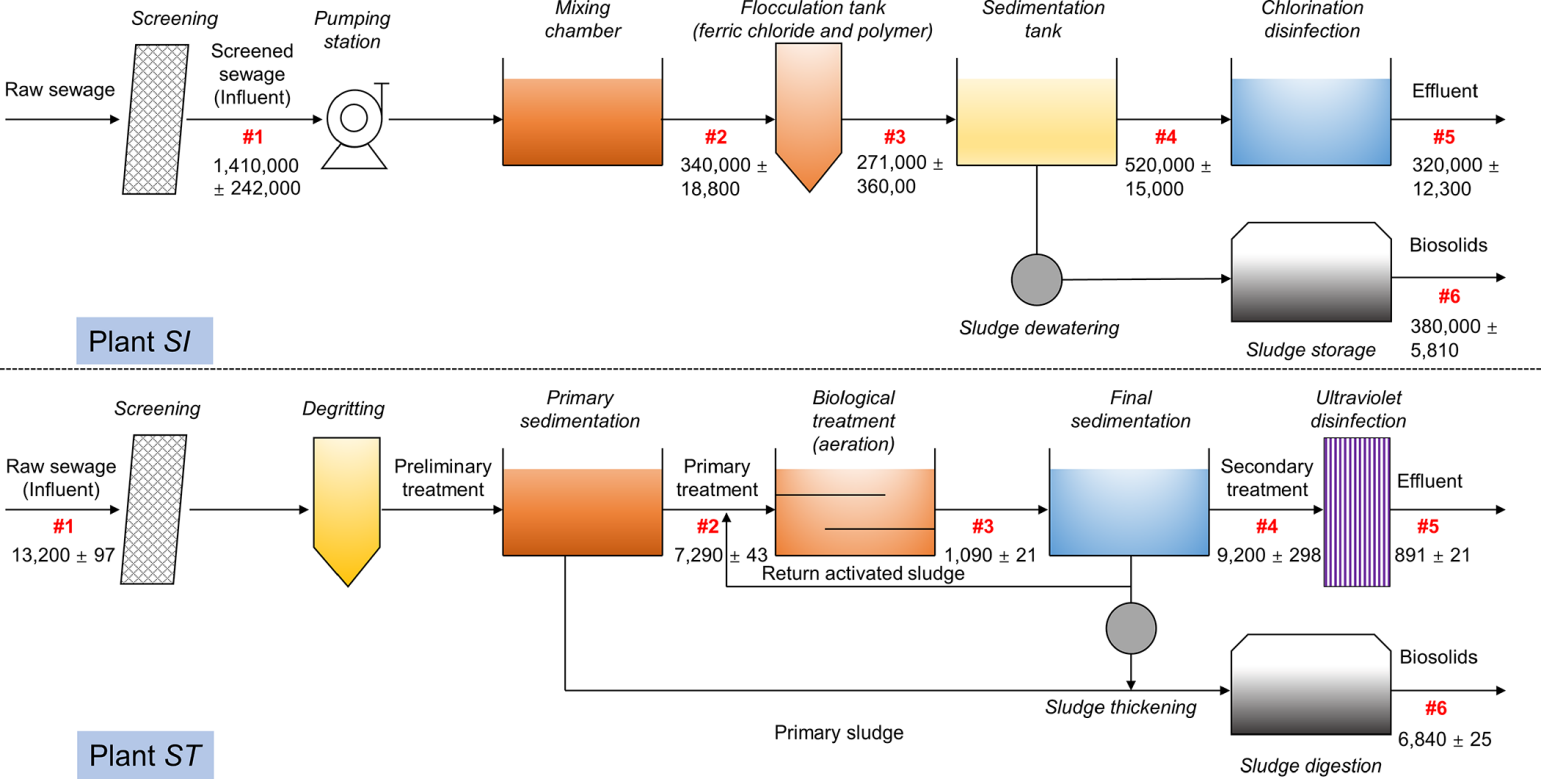
Mass flows (mg/day) of the total PPD-Qs and PPDs in each processing unit of Plants SI (upper) and ST (lower). #1–#5 represent wastewater samples among different processing stages, whereas #6 represents biosolids.

As the processing technologies of WWTPs can greatly influence the efficiency of contaminant elimination, the removal efficiencies for individual PPD-Qs and PPDs among the four WWTPs are displayed in Figure 2. It was found that the median removal efficiencies for PPD-Qs and PPDs ranged from 53.0 to 90.9% and 48.8–76.8%, respectively. The observations suggest that the current processing technologies are effective in reducing a significant amount of these contaminants but not all of them. As the most dominant species of PPD-Qs, 87.3% of 6PPD-Q, and 90.9% of DPPD-Q were eliminated through Hong Kong WWTP treatment. Our results also indicated that secondary treatment associated with A/O (Plant ST) or MBBR (Plant SL) exhibited higher elimination efficiencies for PPD-Qs and PPDs, with median removal efficiencies of 52.0–95.5% (Figure 2B). By contrast, lower removal was observed in the primary treatment plants (i.e., Plant SI and SHW), where median removal efficiencies were determined in the range from −20.5 to 87.0% (Figure 2A). Figure 2D illustrates the processing unit-based removal efficiencies among the four investigated WWTPs. Removal of organic chemicals in primary sedimentation relies on their sorption to the solid phase through a hydraulic retention of approximately 3 h, which is highly associated with the hydrophobicity of the contaminants. During the treatment, PPD-Qs (median level of 20.5%) and PPDs (median level of 74.5%) exhibited moderate to high removal efficiencies. This could be rationalized by the dissimilarities of hydrophobicity between PPD-Qs and PPDs since the quinones with lower *K*_ow_ were more hydrophilic than their parent compounds (Table S1), making them less likely to sorb to the primary sludge. This trend has been observed in IPPD, which with the lowest *K*_ow_ value had the lowest removal percentage among the suite of contaminants. Results from Plants SI and SHW also indicate that the CEPT unit is more effective in removing PPDs (median level of 43.5%) as compared to PPD-Qs (median level of 2.0%). In contrast to the primary and CEPT treatments, the effects of the secondary treatment on the removal of PPD-Qs and PPDs varied. The median removal efficiency for PPD-Qs was determined to be −53.3 to 24.0%. The negative removals of some PPD-Qs can be rationalized by the oxygen-enriched environment in the aeration tank with air or pure oxygen, which may lead to the conversion of PPDs to the corresponding PPD-Qs since all PPDs exhibited positive removal efficiency during this stage (3.6–27.9%). However, this possibility still needs further investigation. Similar findings have been reported in the elimination of pharmaceuticals and personal care products in Spain WWTPs.^39,40^ Among the investigated WWTPs, the effluent undergoes disinfection with either UV irradiation or chlorination prior to discharge into the receiving water. Chlorination with sodium hypochlorite may result in reactions with organic compounds, while UV irradiation with a wavelength of 254 nm may oxidize organic molecules present in water.^41^ The data showed that chlorination treatment led to approximately 23.5% removal of PPD-Qs, while the number greatly increased to 81.5% with UV treatment. Such findings suggest that photo/chemical reactions may occur in the disinfection processes, and UV irradiation is seen as a useful approach for eliminating PPD-Qs from WWTPs. Meanwhile, we performed a mass balance analysis to interrogate the inputs and emissions of PPD-Qs and their parent PPDs in Hong Kong WWTPs. The mass loadings of PPD-Qs in the WWTPs’ influent were estimated at 1,260,000 mg/day, with individual PPD-Qs contributing between 558 to 687,000 mg/day. An evident reduction of ∑PPD-Qs (252,000 mg/day) was observed in the effluent, with DPPD-Q (184,000 mg/day) being the most prominent, followed by 6PPD-Q (66,500 mg/day). In parallel, our results indicated that biosolids endured considerable levels of PPD-Q mass loadings in the four WWTPs (298,000 mg/day), primarily consisting of the contaminants DPPD-Q (288,000 mg/day) and 6PPD-Q (8720 mg/day), respectively. The observation suggested some compositional uniformity of these pollutants in both the effluent and biosolids. It should be noted that the differences between the hydraulic retention time and sludge retention time may affect the mass balance calculations. As compared, the mass loadings of PPDs in the effluent and biosolids were determined to be 68,900 and 90,200 mg/day, respectively, representing 41.5 and 54.2% of their total inputs via the WWTP influent. The percentage mass fluxes of PPDs and PPD-Qs in effluent and biosolids compared to influent are illustrated in Figure S3. It can be seen that the mass outflows of PPDs and PPD-Qs via the effluent and biosolids are inconsistent with the mass inflows via the influent, which suggests that the formation and/or degradation of these contaminants by biotic (e.g., biodegradation in the secondary plants) and abiotic processes (e.g., photolytic degradation in the UV reactors) may occur among the investigated Hong Kong WWTPs. In addition, the specific mass flows of the total PPD-Qs and PPDs among each processing unit were investigated. Significant differences were observed among the WWTPs with different processing technologies and service areas, as shown in Figures 3 and S4. Plant SI exhibited the highest mass flows for the target contaminants, while Plant SL had the lowest. High mass flux percentages of PPD-Qs and PPDs were found in the biosolids of secondary treatment WWTPs (i.e., Plant ST and SL). It was observed that the mixing chamber process in primary treatment plants resulted in an obvious reduction of the mass loadings of PPD-Qs and PPDs (81.9% in Plant SHW and 75.8% in Plant SI), while in the secondary treatment plants, an apparent decrease of the mass flow of PPD-Qs and PPDs was observed in the biological treatment, with reductions of 85.1% in Plant ST and 61.1% in Plant SL. The mass flows (mg/day) of each PPD-Qs and PPDs in different processing units in the investigated Hong Kong WWTPs are summarized in Table S4. Our results suggested that the mass flow in the effluent and biosolids among these studied WWTPs ranged from 113 ± 11 to 320,000 ± 12,300 mg/day and 234 ± 12 to 380,000 ± 5810 mg/d, respectively. A high mass of these pollutants was found to be released into the environment mainly due to large processing flow and insufficient removal.

### 3.3 Environmental Releases of PPD-Qs and PPDs via WWTP Discharges

**Figure 4:**
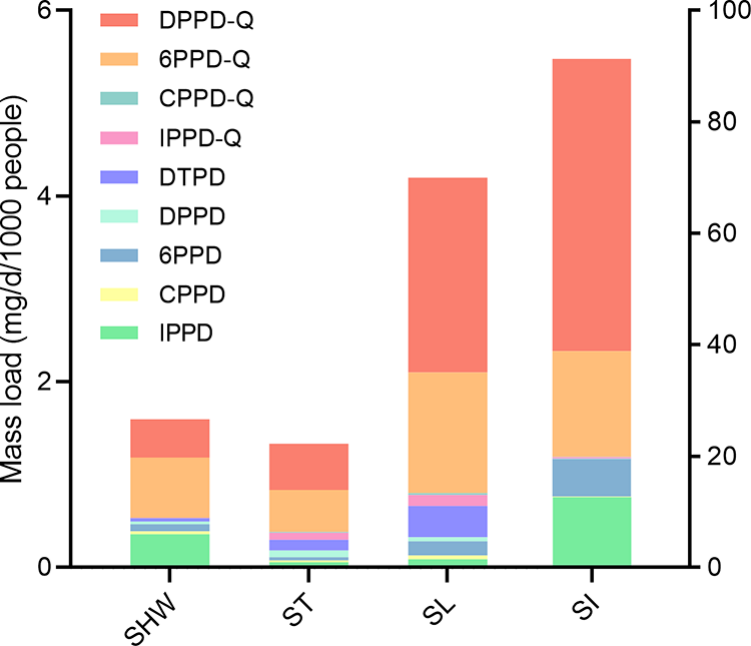
Average daily per capita emission of PPD-Qs and PPDs via effluent in Hong Kong WWTPs. The values of Plant SI are plotted on the right axis.

The average daily per capita emissions of PPD-Qs and PPDs in Hong Kong WWTPs were calculated based on measured concentrations via the effluent. As shown in Figure 4, PPD-Qs and PPDs discharged into the receiving water system from WWTP effluents ranged from 1.34 to 91.3 μg/day/person. A significantly higher environmental release was found for ∑PPD-Qs (1.11–71.7 μg/day/person) compared with that of ∑PPDs (0.259–19.6 μg/day/person). DPPD-Q (0.415–52.5 μg/day/person) exhibited the highest emission values among PPD-Qs, followed by 6PPD-Q (0.454–18.9 μg/day/person) and IPPD-Q (0.039–0.398 μg/day/person). It was noted that PPD-Qs exhibited one to two orders of magnitude higher emission levels than their parent compounds, where IPPD (0.06–12.7 μg/day/person) exhibited the highest emission values among PPDs. In our measurement, high levels of PPD-Qs and PPDs were retained in the biosolids of WWTPs. According to the Environmental Protection Department (EPD) of Hong Kong, around 1,800 tons of biosolids are generated from WWTPs every day and most of them are incinerated or disposed of in landfills. Around 6% of the total amount of biosolids generated in Hong Kong WWTPs was recycled as a soil conditioner. Therefore, we have calculated the daily per capita emissions of PPD-Qs and PPDs via the recycled biosolids in the investigated WWTPs, where a comparable release level was observed for ∑PPD-Qs (0.040–1.49 μg/day per person) and ∑PPDs (0.040–1.53 μg/day/person). However, it is worth noting that the effluent takes a dominant role in the environmental release of PPD-Qs and PPDs to Hong Kong citizens, in comparison via the recycled biosolids in the investigated WWTPs even at a worst-case scenario. As a consequence, these results indicate that PPD-Qs exhibit greater discharges than their parent PPDs through the effluent of Hong Kong WWTPs, which may pose a significant risk of ecological hazards and consequences in aquatic environments.

## 4 Environmental Implications

This study represents the first investigation of PPD-Q occurrence in municipal WWTPs in Hong Kong. The mass balance, removal, and environmental release of these emerging contaminants along with their parent compound PPDs were investigated. Our results demonstrated that current processing technologies cannot completely eliminate PPDs and PPD-Qs, where PPD-Qs exhibited higher emission levels than their parent PPDs in both the effluent and biosolids. Among the suite of anthropogenic contaminants, a considerable level of 6PPD-Q, which is highly toxic to coho salmon, rainbow trout, and brook trout, has been detected in the influent of Hong Kong WWTPs. A previous study by Hiki et al. has reported that 6PPD-Q was more stable than its parent compound 6PPD in dechlorinated tap water.^42^ In line with this finding, our research further supports that all five PPD-Qs exhibit higher half-lives than their corresponding PPDs. It can be anticipated that PPD-Qs would be retained in aqueous systems for longer periods of time and cause long-lasting environmental consequences to aquatic ecosystems. By comparing the elimination efficacy of these contaminants in each unit, we found that the secondary treatment plants exhibit removal efficiencies for PPDs higher than those of the primary treatment plants. Meanwhile, it is found that primary treatment, CEPT, and UV disinfection contribute to most of the removal of PPDs among the investigated WWTPs, whereas PPD-Qs are mainly eliminated through UV disinfection, primary treatment, and chlorination processes. In addition, our estimate of the average daily emissions of PPD-Qs and PPDs varied among the Hong Kong WWTPs, with DPPD-Q and 6PPD-Q identified as the dominant species in WWTP discharges.

In 2022, the U.S. Department of Toxic Substances Control (DTSC) and Tire Manufacturers Association jointly initiated rulemaking to list motor vehicle tires containing 6PPD as a priority product with a major concern for the significant adverse impacts of 6PPD-Q to aquatic organisms, especially for two populations of coho salmon. However, for other PPDs, the existing alternatives of 6PPD, there is a paucity of research on evaluating the ecotoxicity of their quinone transformation products PPD-Qs, which have been evidently detected in the influent and effluent in Hong Kong WWTPs and sediments across estuaries, coasts, and deep-sea regions of the South China Sea.^24^ In view of these findings, further research is needed to interrogate the potential ecological hazards and health risks of 6PPD-Q and other PPD-Qs from wastewater discharge on marine species in coastal areas, especially at the environmental level. As of now, there is ample evidence indicating that a wide range of rubber products and products related to human activities such as tire rubber, crumb rubber, E-waste recycling, and elastomeric consumer products contain PPDs and PPD-Qs.^23,43^ It is of particular importance to assess the sources and proportions of these contaminants from such rubber products, with various anthropogenic activities, including population density and traffic volume being taken into account.

## Supporting Information Available

Half-life measurement, data calculation, land utilization, effluent discharge direction and processing characteristics of each investigated WWTP, optimized MRM parameters, recoveries, LOQs and LODs of the analytes, percentage mass flux of PPDs and PPD-Qs in the effluent, and biosolids and mass flows of PPD-Qs and PPDs in plants SHW and SL (PDF)
