# Supplementary material for: Occurrence and Fate of Substituted p-Phenylenediamine-Derived Quinones in Hong Kong Wastewater Treatment Plants
Source: Environ Sci Technol. 2023 Oct 5;57(41):15635–43. doi: 10.1021/acs.est.3c03758 (PMC10586368; doi:10.1021/acs.est.3c03758)
Supplement: Supplementary file 1 — es3c03758_si_001.pdf [file es3c03758_si_001.pdf]

Supporting Information for

Occurrence and Fate of Substituted *p*-Phenylenediamine Derived Quinones in Hong Kong Wastewater Treatment Plants

Guodong Cao[1], Wei Wang[1], Jing Zhang, Pengfei Wu, Han Qiao, Huankai Li, Gefei Huang, Zhu Yang, Zongwei Cai*

State Key Laboratory of Environmental and Biological Analysis, Department of Chemistry, Hong Kong Baptist University, Hong Kong SAR 999077, China

Number of pages: 12

Number of figures: 4

Number of tables: 4

Contents

Text S1. Determination of half-lives of PPD-Qs and PPDs in dechlorinated tap water. S2

Text S2. Data calculation...S2

Table S1. Information on each analyte of PPD-Qs S4

Table S2. The main process characteristics of each investigated WWTP...............................S5

Table S3. Optimized MRM parameters, recoveries, LOQs and LODs of the analytes............S6

Table S4. Mass flows of PPD-Qs and PPDs in each processing unit......................................S7

Figure S1. Concentrations versus time plots for the estimation of the half-life of PPD-Qs in dechlorinated tap water with one-phase decay model. S8

Figure S2. Land utilization of Hong Kong and servicing areas of each WWTPs S9

Figure S3. Percentage mass flux of PPDs and PPD-Qs in effluent and biosolids..................S10

Figure S4. Mass flows of PPD-Qs and PPDs in Plants SHW and SL S11

References S12

* Corresponding author:

E-mail addresses: zwcai@hkbu.edu.hk (Z. Cai).

[1] Both authors contribute equally to this work.

Text S1. Determination of half-lives of PPD-Qs and PPDs in dechlorinated tap water.

0.2 mL of each stock solution of each PPD-Qs and PPDs dissolved in acetonitrile (1.0 mg/L) was added into amber glass bottle and blow dry with nitrogen, which was placed within a fume hood for an additional 30 s to let excess organic solvent vapor evacuate as described by Hu et al[1]. After drying, 20 mL of dechlorinated tap water (pH 7.55, conductivity 197.1 μS/cm) was added into the bottles, followed by a 15 min ultrasonication, and then transferred onto a shaker (HiPoint 500SRC) at 70 rpm/min maintained at 20 ± 1 °C for the stability test. For the MS analysis, 160 μL of the solution was sampled and mixed with 40 μL of solution of internal standard (50 ppb 6PPD-Q-d_5 in acetonitrile). The concentrations of PPD-Qs and PPDs were measured at different time points using the HPLC-MS/MS method as described in Section 2.4. The stability test was maintained for one month. The half-lives of the PPD-Qs and PPDs were calculated based on one phase decay exponential model. The results were described in Table S1. It was noted that the half-life of 6PPD-Q measured in this study was longer than the previous report by Hiki et al[2]. Several factors, such as the temperature during the time-course experiment and the use of solvent to prepare 6PPD-Q solutions may contribute to the measured difference between the two studies.

Text S2. Data calculation.

The removal efficiency and mass balance calculation in this study are in reference to the former studies [3, 4]. The ability of the WWTPs to remove PPD and PPD-Qs was determined by calculating the removal efficiency (RE, %) between their concentrations of them in the influent and final effluent wastewater of the WWTPs as shown in Eq (1):

$$RE\ (\%) = \frac{(C_{inf} - C_{eff}) \times 100\%}{C_{inf}} \tag{1}$$

where C_{inf} and C_{eff} are the concentrations of PPD antioxidants and PPD-Qs detected at the influent and the effluent of each WWTPs, respectively. When non-detects were present in both influent and final effluent, the computation was unavailable. When influent was detected and the final effluent was non-detected, the non-detects were substituted with half the LOQ.

Mass flux (MF, mg/day) was estimated by multiplying contaminant concentrations with volumetric flow rates for each processing unit, and with production rate for biosolids as shown in Eq (2)-(3):

$$MF_{was} = C_{was} \times Q_{was} \tag{2}$$

$$MF_{bio} = C_{bio} \times Q_{bio} \tag{3}$$

where C_{was} and C_{bio} are the concentrations of PPD antioxidants and PPD-Qs detected at each processing unit of wastewater (mg/L) and in biosolids (mg/g d.w.), respectively. Q_{was} and Q_{bio} are the corresponding daily flow rate of wastewater (L/day) and actual daily production of sludge (g/day), respectively.

Mass balance calculations were conducted using contaminant mass flux in wastewater from each processing unit, and biosolids to quantify the removal mechanisms via degradation and/or sorption as shown in Eq (4):

$$MF_{inf} = MF_{eff} + MF_{bio} + MF_{for} - MF_{deg} \tag{4}$$

where MF_{inf}, MF_{eff} and MF_{bio} are the mass flux of PPD antioxidants and PPD-Qs in the influent and effluent of wastewater (mg/day) and biosolids (mg/day), respectively. MF_{for} and MF_{deg} represent the formation and/or degradation by biotic (e.g., biodegradation in the secondary plants) and abiotic processes (e.g., photolytic degradation in the UV reactors), respectively.

To better understand the environmental release rate of these PPDs and PPD-Qs, their back-estimated per-capita environmental emission mass load (EML, μg/day/person) in distinct served by each selected WWTP was calculated by Eq (5)-(6), where Pop is the population that is served by the corresponding WWTPs.

$$EML_{eff} = \frac{MF_{eff}}{Pop} \tag{5}$$

$$EML_{bio} = \frac{MF_{bio}}{Pop} \tag{6}$$

Table S1. Information on analyte name, abbreviations, CAS No, structure and physicochemical properties calculated by EPI Suites[5].

Compound name	Abbreviation	CAS No.	Structure	Log K_{ow}	Water solubility (mg/L, 25 ℃)	Half-life
N-isopropyl-m'-phenyl-p-phenylenediamine	IPPD	101-72-4	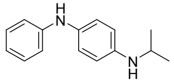	3.28	50.32	4.1 h
N-phenyl-N'-cyclohexyl-p-phenylenediamine	CPPD	101-87-1	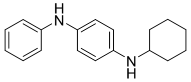	4.93	1.277	6.0 h
N-1, 3-dimethylbutyl-n'-phenyl-p-phenylenediamine	6PPD	793-24-8	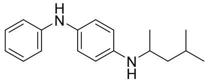	4.64	2.083	4.5 h
N,N'-diphenyl-p-phenylenediamine	DPPD	74-31-7	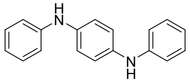	4.47	2.8262	45.3 h
N,N''-bis(methylphenyl)-1,4-benzenediamine	DTPD	15017-02-4	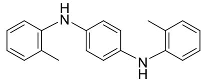	5.13	0.5914	32.5 h
2-anilino5-[(4-methylpentan-2-yl)amino]cyclohexa-2,5diene-1,4-dione	6PPD-Q	2754428-18-5	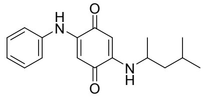	3.94	56.93	194.1 h
2-(cyclohexylamino)-5-(phenylamino)cyclohexa-2,5-diene-1,4-dione	CPPD-Q	68054-78-4	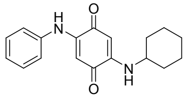	3.46	15.38	83.4 h
2,5-bis(phenylamino)cyclohexa-2,5-diene-1,4-dione	DPPD-Q	3421-08-7	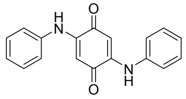	3.98	51.34	157.8 h
2,5-bis(o-tolylamino)cyclohexa-2,5-diene-1,4-dione	DTPD-Q	1005173-46-5	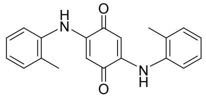	4.56	1.225	251.9 h
2-(isopropylamino)-5-(phenylamino)cyclohexa-2,5-diene-1,4-dione	IPPD-Q	68054-73-9	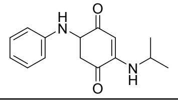	2.58	1400	632.4 h

Table S2. The main process characteristics of each WWTP. Data were obtained from Drainage Department of Hong Kong.

WWTP characteristics	Stonecutters Island (SI)	Siu Ho Wan (SHW)	Sha Tin (ST)	Stanley (SL)
Process type	Chemically enhanced primary treatment (CEPT)	Chemically enhanced primary treatment (CEPT)	Secondary activated sludge, anaerobic/oxic (A/O)	Secondary activated sludge, moving-bed biofilm reactor (MBBR)
Average flow (m^3/d)	1,800,000	50,000	260,000	9,000
Population served	3,500,000	200,000	650,000	27,000
Significant industries	Industrial, domestic sewage, hospital	domestic sewage, amusement park	domestic sewage	domestic sewage
Disinfection	Chlorination	UV	UV	Chlorination
Solid treatment	Dewatering	Dewatering	Anaerobic digestion Dewatering	Dewatering
Moisture content (%)	68	70	70	70
Receiving water	South China Sea	South China Sea	South China Sea	South China Sea
Sludge production (ton/day)	1200	33	120	4

Table S3. Optimized MRM parameters, recoveries, LOQs and LODs of the analytes in influent, other (wastewater samples besides influent) and biosolids.

Compound	Precursor ion (m/z)	Quantifier product ion (m/z)	Collision energy (V)	Qualifier product ion (m/z)	Wastewater recovery (%)	Biosolids recovery (%)	Influent LOQ/LOD (ng/L)	Other LOQ/LOD (ng/L)	Biosolids LOQ/LOD (ng/g)
IPPD	227.2	184.1	26	212.1<br>168.1	78±11	71±6	0.03/<br>0.009	0.01/<br>0.004	0.07/<br>0.021
CPPD	267.2	185.1	22	223.1<br>130.1	78±14	77±10	0.07/<br>0.021	0.04/<br>0.011	0.18/<br>0.053
6PPD	269.2	93.1	32	184.1<br>212.1	75±16	80±10	0.12/<br>0.037	0.06/<br>0.018	0.31/<br>0.092
DPPD	261.1	184.1	26	169.1<br>107.1	113±22	111±11	0.14/<br>0.042	0.07/<br>0.021	0.35/<br>0.105
DTPD	289.2	198.1	23	183.1<br>106.1	104±28	76±35	0.09/<br>0.026	0.04/<br>0.013	0.22/<br>0.065
IPPD-Q	257.1	187.1	24	215.1<br>172.1	92±7	82±1	0.10/<br>0.031	0.05/<br>0.015	0.26/<br>0.077
CPPD-Q	297.2	187.1	28	215.1<br>98.1	100±1	95±5	0.10/<br>0.030	0.05/<br>0.015	0.25/<br>0.074
6PPD-Q	299.2	241.1	26	215.1<br>187.1	95±1	99±3	0.02/<br>0.005	0.01/<br>0.002	0.04/<br>0.012
DPPD-Q	291.1	263.1	20	235.1<br>144.1	85±5	94±5	0.04/<br>0.013	0.02/<br>0.007	0.11/<br>0.033
DTPD-Q	319.1	184.1	27	212.1<br>301.1	96±2	104±1	0.06/<br>0.017	0.03/<br>0.009	0.14/<br>0.043

Table S4. Mass flows (mg/day) of the total PPD-Qs and PPDs in each processing unit in the investigated Hong Kong WWTPs. #1-#5 represent wastewater samples from influent to effluent, whereas #6 represents biosolids.

Unit	IPPD	CPPD	6PPD	DPPD	DTPD	IPPD-Q	CPPD-Q	6PPD-Q	DPPD-Q
**#1 (SI)**	59100	655	96200	720	0	1740	468	685000	561000
**#2 (SI)**	33700	1440	225000	353	0	3550	212	45300	30600
**#3 (SI)**	15000	418	219000	176	0	3520	277	3160	28500
**#4 (SI)**	15200	346	151000	223	0	3810	443	90300	258000
**#5 (SI)**	44300	342	23600	349	0	864	198	66100	184000
**6# (SI)**	2270	708	83200	2280	864	138	564	7790	282000
**#1 (SHW)**	41.8	14.6	63	20	0	25.2	0	1030	7550
**#2 (SHW)**	124	15.7	132	7	0	20.8	0	537	746
**#3 (SHW)**	128	7.9	165	10.3	0	6.2	0	631	816
**#4 (SHW)**	314	7.9	18.3	10.3	0	27.4	0	1310	2960
**#5 (SHW)**	71.5	6.5	15.1	11	0	7.8	0	130	82.9
**#6 (SHW)**	22.1	26.9	296	25.7	17.8	6.11	24.4	86.5	616
**#1 (SL)**	6.19	7.09	17.2	10.8	11.3	22.3	1.8	748	731
**#2 (SL)**	2.29	1.16	6.02	2.47	1.34	8.64	1.62	75	127
**#3 (SL)**	1.72	1.16	3.63	2.47	1.34	7.02	1.18	23.9	45.3
**#4 (SL)**	0.87	1.8	4.08	1.44	1.56	5.64	2.04	58.9	145
**#5 (SL)**	2.34	1.13	4.09	1.26	1.34	10.7	0.489	35	56.8
**#6 (SL)**	1.00	2.76	9.96	1.98	2.12	1.2	9.84	24.6	181
**#1 (ST)**	2920	127	5960	192	185	246	88.4	485	2980
**#2 (ST)**	565	30.5	1280	81	78	66.9	159	785	4250
**#3 (ST)**	24.3	28.9	99.4	63.6	62.4	144	32.9	315	317
**#4 (ST)**	325	32.7	1180	77.3	80.2	206	135	1930	5230
**#5 (ST)**	35.8	12.4	22.1	50.7	47.5	96.2	5.85	295	325
**#6 (ST)**	34.8	61.2	264	61.8	64.8	22.2	187	823	5320

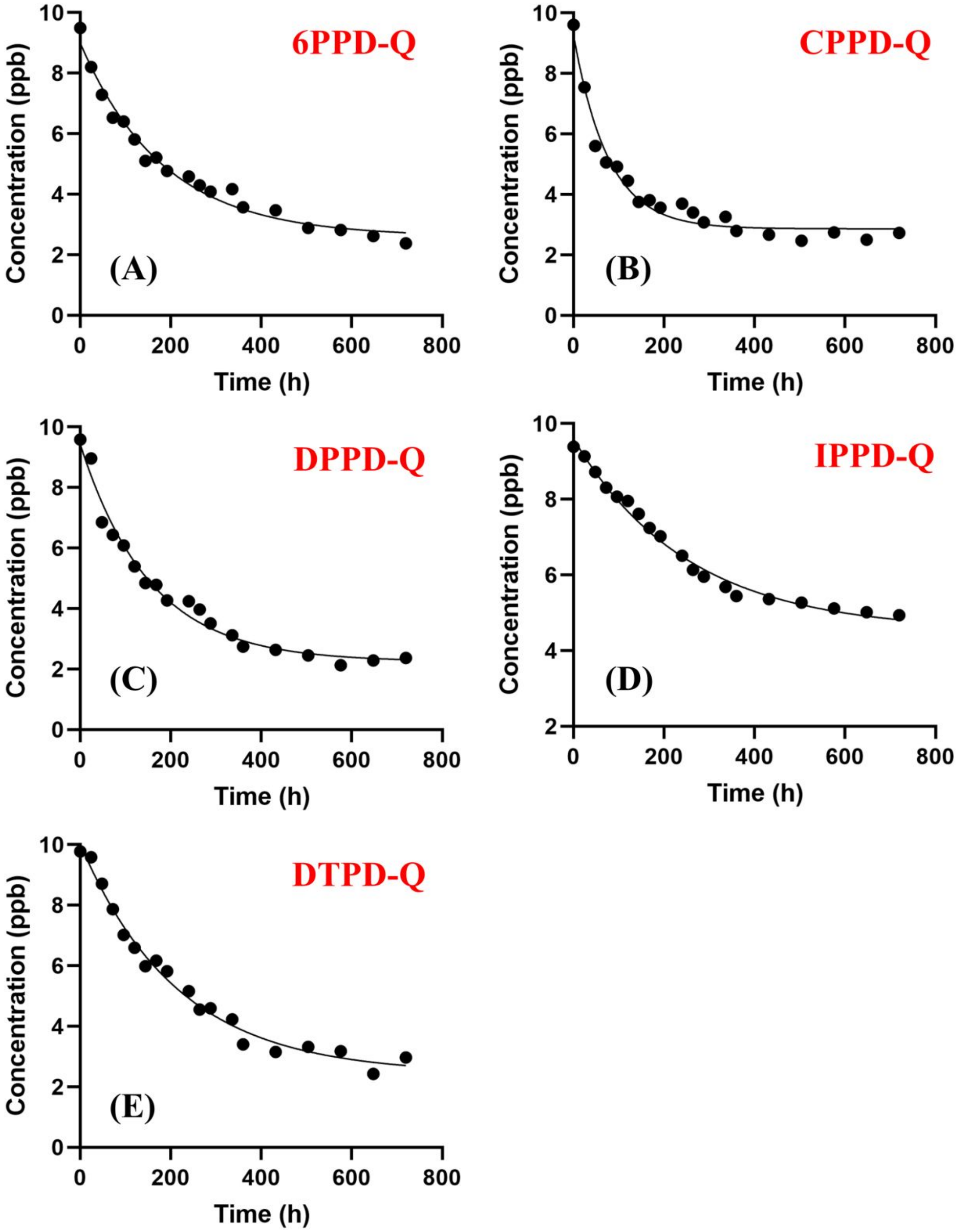

Figure S1. Concentrations versus time plots for the estimation of the half-life of PPD-Qs in dechlorinated tap water using one-phase exponential decay model.

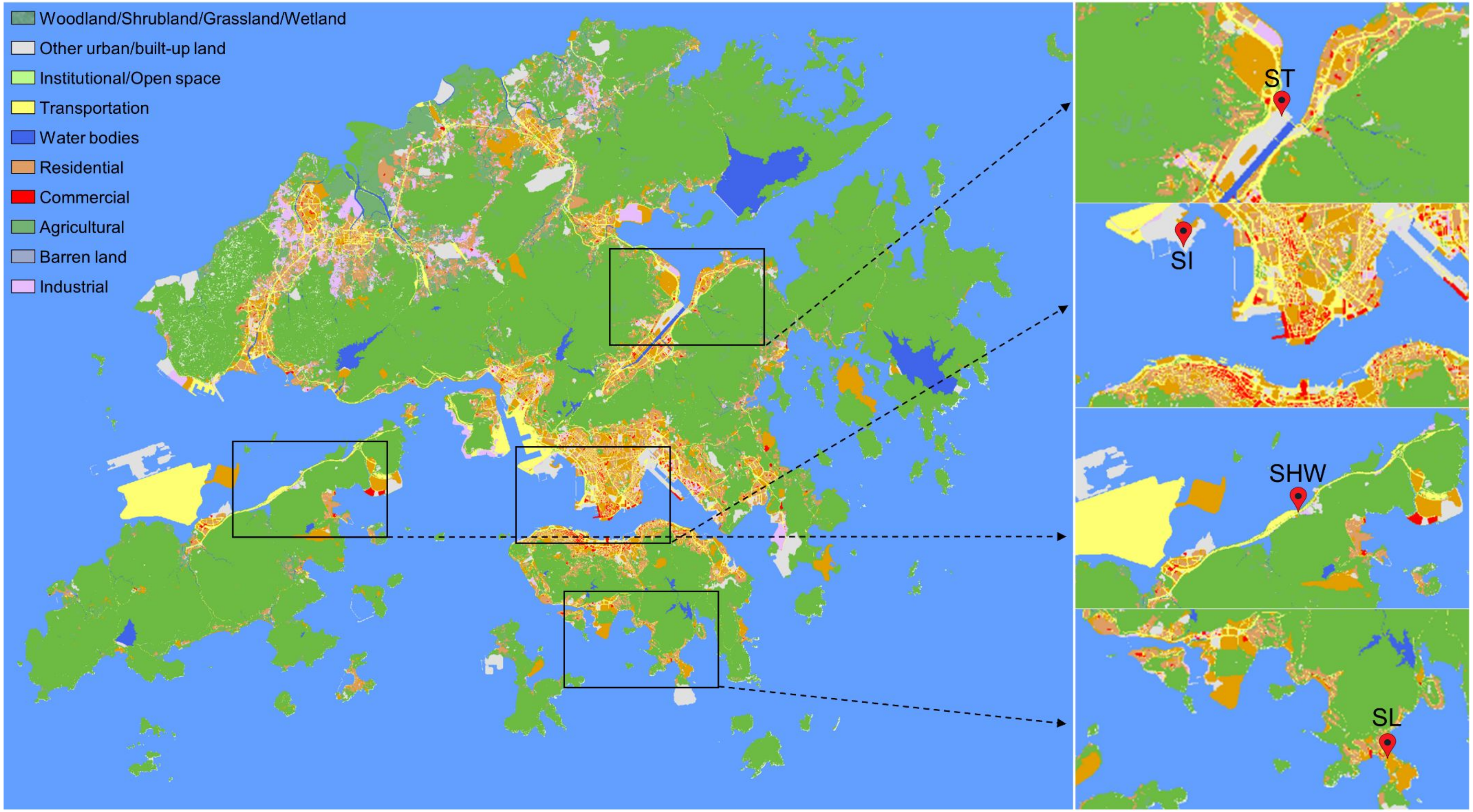

Figure S2. Land utilization of Hong Kong and servicing areas of each investigated WWTP. Data were obtained from Planning Department of Hong Kong.

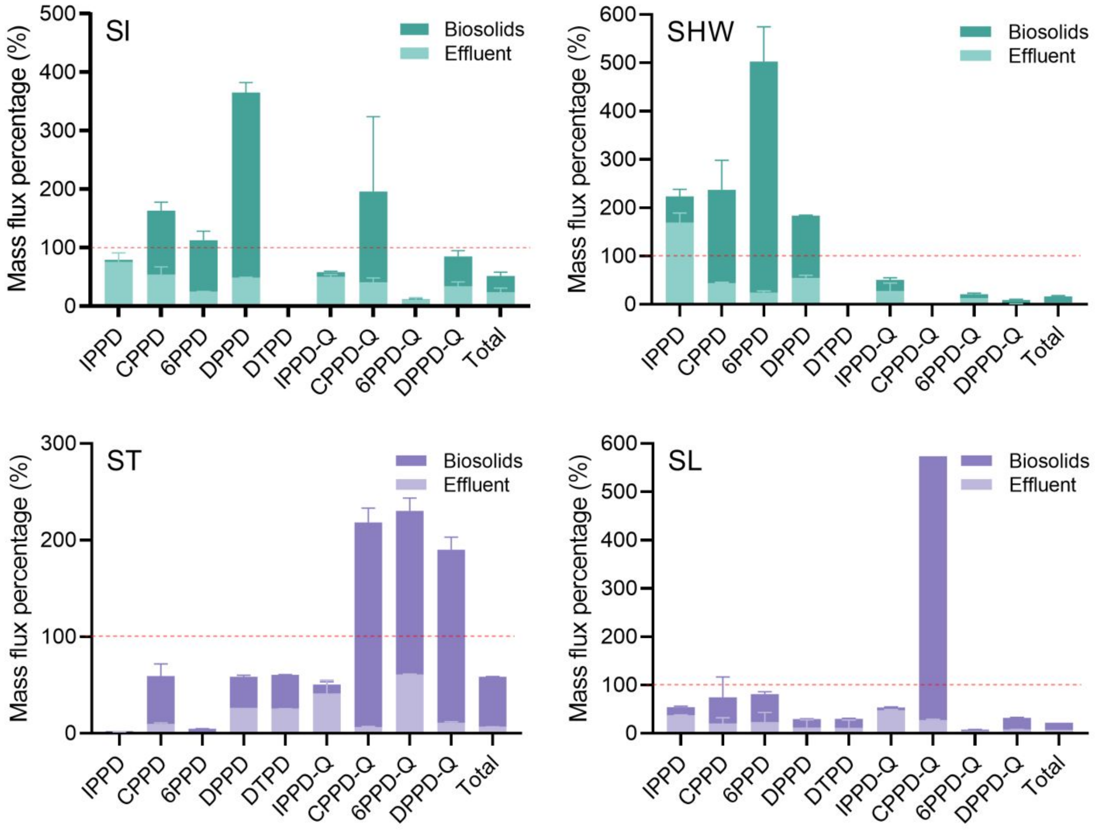

Figure S3. Percentage mass flux of PPDs and PPD-Qs in effluent and biosolids compared to the influent. Data is illustrated as mean ± S.D. and the fraction of effluent and biosolids are stacked. SI and SHW in green represent WWTPs with primary treatment, ST and SL in purple represent WWTPs with secondary treatment. (According to the criteria in Section 2.1, DTPD and DTPD-Q with detection rates less than 30% were not shown in the Figure).

.

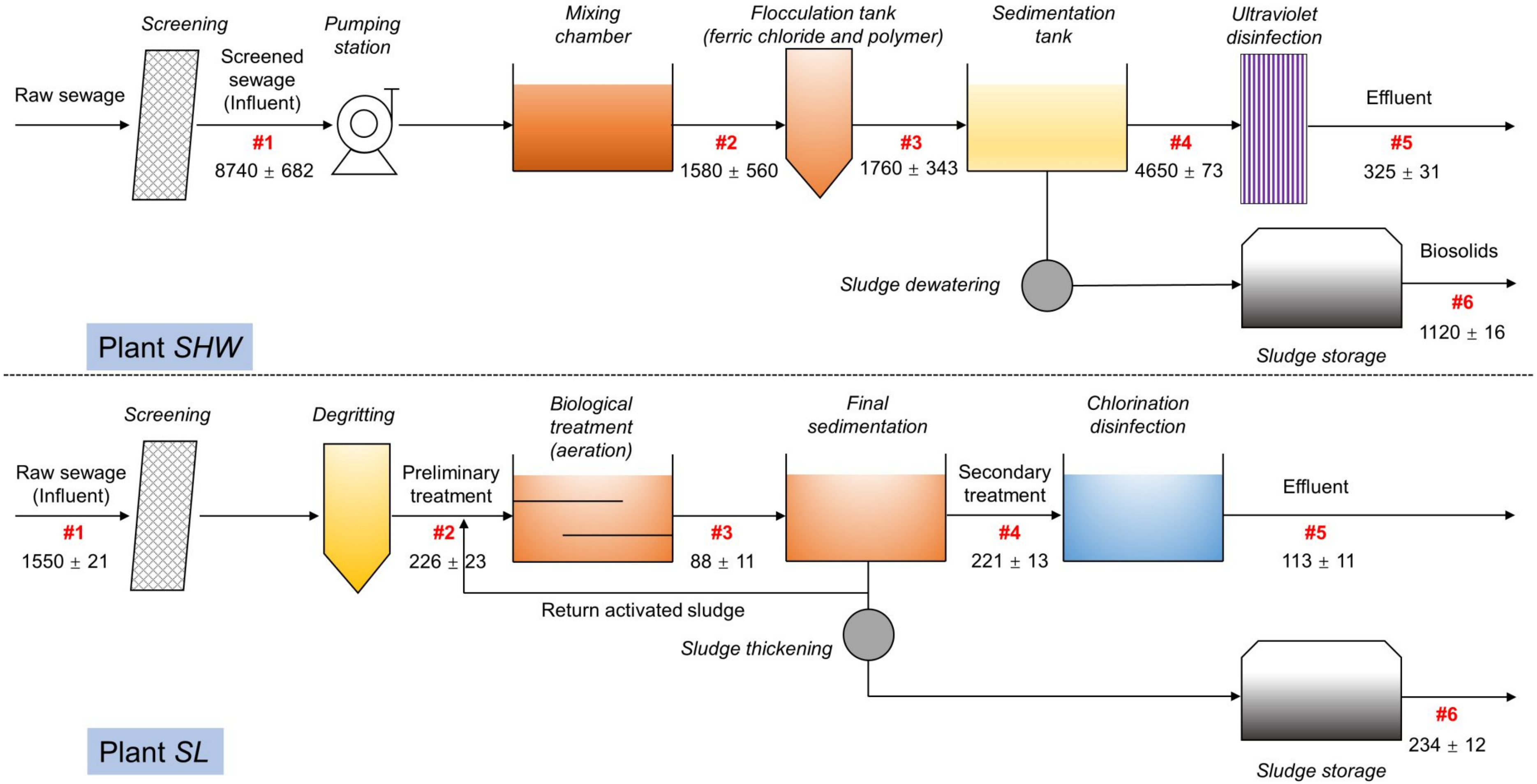

Figure S4. Mass flows (mg/day) of PPD-Qs and PPDs in each processing unit of Plants SHW (upper) and SL (lower). #1-#5 represent wastewater samples among different processing stages, whereas #6 represents biosolids.

References

1. Hu, X.; Zhao, H. N.; Tian, Z.; Peter, K. T.; Dodd, M. C.; Kolodziej, E. P., Chemical characteristics, leaching, and stability of the ubiquitous tire rubber-derived toxicant 6PPD-quinone. *Environmental Science: Processes & Impacts* **2023**.
2. Hiki, K.; Asahina, K.; Kato, K.; Yamagishi, T.; Omagari, R.; Iwasaki, Y.; Watanabe, H.; Yamamoto, H., Acute toxicity of a tire rubber-derived chemical, 6PPD quinone, to freshwater fish and crustacean species. *Environmental Science & Technology Letters* **2021,** *8*, (9), 779-784.
3. Zhang, Z. F.; Zhang, X.; Zhang, X.; Sverko, E.; Smyth, S. A.; Li, Y. F., Diphenylamine Antioxidants in wastewater influent, effluent, biosolids and landfill leachate: Contribution to environmental releases. *Water Res.* **2021,** *189*, 116602.
4. Yao, L.; Chen, Z. Y.; Dou, W. Y.; Yao, Z. K.; Duan, X. C.; Chen, Z. F.; Zhang, L. J.; Nong, Y. J.; Zhao, J. L.; Ying, G. G., Occurrence, removal and mass loads of antiviral drugs in seven wastewater treatment plants with various treatment processes. *Water Res* **2021,** *207*, 117803.
5. Challis, J.; Popick, H.; Prajapati, S.; Harder, P.; Giesy, J.; McPhedran, K.; Brinkmann, M., Occurrences of tire rubber-derived contaminants in cold-climate urban runoff. *Environmental Science & Technology Letters* **2021,** *8*, (11), 961-967.
